# Effect of menstrual cycle phase and hormonal treatments on evaluation of tubal patency in baboons

**DOI:** 10.1111/jmp.12317

**Published:** 2017-10-24

**Authors:** Jeffrey T. Jensen, Carol Hanna, Emily Mishler, Jeong Y. Lim, Ov D. Slayden

**Affiliations:** ^1^ Department of Obstetrics & Gynecology Oregon Health & Science University (OHSU) Portland OR USA; ^2^ Division of Reproductive and Developmental Sciences Oregon National Primate Research Center Beaverton OR USA

**Keywords:** baboon, estradiol, fallopian tubes, hysterosalpingography

## Abstract

**Background:**

We evaluated whether menstrual cycle phase influences the assessment of tubal patency by hysterosalpingography (HSG) in baboons.

**Methods:**

Retrospective analysis of baseline tubal patency studies and serum estradiol (E_2_) and progesterone (P4) values obtained from female baboons used as models for development of non‐surgical permanent contraception in women. The main outcome measure was bilateral tubal patency (BTP) in relationship with estradiol level.

**Results:**

Female baboons (n = 110) underwent a single (n = 81), two (n = 26), or three (n = 3) HSG examinations. In 33/142 (23%) HSG examinations, one or both tubes showed functional occlusion (FO). The median E_2_ in studies with BTP (49 pg/mL) was significantly higher than in those studies with FO (32 pg/mL,* P* = .005). Among 18 animals with repeat examinations where serum E_2_ changed from <60 to ≥ 60 pg/mL, 13 results changed from FO to BTP (*P* = .0001). No sets showed a change from BTP to FO with an increase in estradiol.

**Conclusion:**

In baboons, functional occlusion of the fallopian tube is associated with low estradiol levels, supporting a role for estrogen‐mediated relaxation of the utero‐tubal junction.

## 1 INTRODUCTION

The American College of Radiology recommends performing evaluation of tubal patency by hysterosalpingogram (HSG) during the early proliferative phase of the menstrual cycle.^1^ This timing prevents inadvertent disruption of a luteal phase pregnancy and may offer other advantages to imaging. More recently, HSG evaluation has been required as a verification step after transcervical hysteroscopic permanent contraception (PC) procedures. While women undergoing infertility evaluation generally are not using hormonal contraceptives, patients are required to continue using a contraception method after hysteroscopic PC until verification of tubal occlusion.

Functional tubal occlusion (FO) is the term used to describe a false‐positive assessment of tubal occlusion at the utero‐tubal junction (UTJ) observed during HSG that is not explained by a true anatomic blockade or the presence of an obstructing material.^2^ FO likely occurs due to muscular spasm at the UTJ that may be cycle dependent.^3^ Few studies have evaluated whether FO is influenced by menstrual cycle timing or hormonal therapy. Lindhal and Helander performed HSG in 511 women of proven fertility and found the highest rates of tubal occlusion during the follicular (proliferative) phase and recommended the early luteal (secretory) phase as the best stage for tubal evaluation.^4^

Menstrual cycle timing and treatments that relax the uterine musculature may also impact the success with non‐surgical permanent contraception techniques such as quinacrine sterilization (QS) or hysteroscopic placement of Essure^™^ microinserts. In a review of QS efficacy, Sokal et al^5^ found that pre‐treatment with papaverine, a uterine relaxant, was associated with a lower failure rate. Sinha found that secretory phase timing reduced the success of placement of Essure^™^ microinserts.^6^ In support of research evaluating non‐surgical approaches to PC, we performed HSGs in female baboons prior to other transcervical procedures.^7^, ^8^ During the course of these studies, we noted differences in the success of our tubal imaging based on cycle timing and hormonal treatment. To explore this hypothesis, we conducted a descriptive retrospective reanalysis of these baseline data to evaluate the association of the hormonal milieu and tubal patency. Here, we present descriptive results from this series, focusing on the correlation of bilateral tubal patency with serum estradiol.

## 2 MATERIALS AND METHODS

### 2.1 Humane care guidelines

Animal husbandry provided by the Southwest National Primate Research Center (SNPRC) and the Oregon National Primate Research Center (ONPRC) are in accord with the National Institutes of Health (NIH) Guidelines for Care and Use of Laboratory Animals and meet or exceed all standards of the Public Health Service's “Policy on the Humane Care and Use of Laboratory Animals.” The SNPRC and ONPRC Institutional Animal Care and Use Committees reviewed and approved all study protocols prior to initiation of the research.

### 2.2 Animals and treatments

Between October 2012 and November 2016, we completed several studies investigating approaches to non‐surgical permanent contraception in women at SNPRC and ONPRC using the baboon model. Results from some of these studies have previously been reported and others are ongoing.^7^, ^8^ We performed one or more baseline hysterosalpingogram procedures and serum sampling for estradiol and progesterone prior to the investigational treatment in 110 healthy adult (ages 5—16 years) female baboons (Papio anubis n = 62, [SNPRC]; Papio hamadryas n = 48, [ONPRC]). Clinical veterinary staff at SNPRC and ONPRC confirmed health status through review of medical histories and performing screening physical examinations. A subset of animals underwent either two (n = 26) or three (n = 3) repeat baseline examinations. We determined menstrual cycle stage by measurement of serum estradiol (E2) and progesterone (P). Samples were obtained by femoral venipuncture on the day of each procedure, and serum was frozen at −80°C until shipped to ONPRC for analysis. The E2 and P assays were carried out with a specific electrochemoluminescent Roche Elecsys 2010 analyzer (F. Hoffmann‐La Roche Ltd, Basel, Switzerland) by the Endocrine Services Core Laboratory, Oregon National Primate Research Center (ONPRC) as previously described.^9^

As baboons undergo significant sexual skin swelling during natural menstrual cycles,^10^ we used a variety of strategies to control cycle stage to facilitate examination. While most animals underwent the HSG procedure during a natural menstrual cycle, in some protocols, animals received a one‐time pre‐treatment intramuscular injection of either depomedroxyprogesterone acetate (DMPA, 3.5 mg/kg, Pharma & Upjohn, New York, NY, USA, n = 10), depo leuprolide acetate (Eligard, 1.88 mg (approximately 90 μg/kg), Tolmar Pharmaceuticals, Fort Collins, CO, USA, n = 7), or Antide (3 mg/kg, n = 6) or a combined oral contraceptive (COC, ethinyl estradiol 30 μg/levonorgestrel 150 μg, Teva Pharmaceuticals, Petach Tikva, Israel, n = 28) prior to the HSG procedure. All of the GnRH against/antagonist‐treated females and three of the DMPA‐treated animals received drug within 1 week of the HSG. COC‐treated females received one pill daily for 18‐21 days, followed by a pill‐free interval of 1‐8 days prior to the HSG.

### 2.3 Hysterosalpingogram procedures

Females underwent a hysterosalpingogram (HSG) procedure to confirm tubal patency as previously described.^8^ The same investigator performed all of the procedures. Briefly, under transabdominal ultrasound guidance, we passed a small silicone HSG balloon catheter (model J‐CHSG‐503000, Cook, Bloomington, IN, USA) through the cervix into the uterine cavity of anesthetized animals. At SNPRC, a series of digital radiographs were obtained to evaluate tubal patency (unilateral patent, bilateral patent and bilateral non‐patent) following the infusion of small aliquots (1‐3 mL) of radiopaque contrast (Isovue^®^, iopamidol injection, Bracco Diagnostics, Monroe Township, NJ, USA). At ONPRC, we followed the same approach but used continuous fluoroscopy (GE OEC 9600 ESP C‐ARM, GE Healthcare, Wauwatosa, WI, USA). We also recorded the amount of contrast administered (mL), the presence of vascular uptake of contrast scored as absent (no vessels), minimal (small vessels only), or substantial (extravasation of contrast into the iliac or renal vasculature) and subjectively evaluated the difficulty of the examination (easy, moderate and difficult) and pressure needed to administer contrast (low, moderate and high). A digital archive of images allowed us to verify impressions recorded on the day of procedure. We categorized tubal imaging as bilateral tubal patency (BTP) when we observed clear fill and spill of both tubes and functional occlusion (FO) when one or both tubes were blocked at the utero‐tubal junction (UTJ).

### 2.4 Verification of tubal patency

In cases where we could not confirm bilateral tubal patency by HSG, we evaluated patency ex vivo and histologically as previously described.^8^

### 2.5 Data analysis

This study presents descriptive data on tubal patency from a series of examinations carried out to evaluate approaches to non‐surgical permanent contraception carried out without an a priori hypothesis or formal power analysis. We report descriptive statistics of tubal patency results. As estradiol concentrations were not normally distributed, we compared the median level of E_2_ in examinations with BTP to those with FO using the Mann‐Whitney U‐test. We stratified the studies according to estradiol concentration (E_2_ ≥ 60, E_2_ < 60 pg/mL) and compared the proportion of examinations with BTP using Fisher's exact test, and further evaluated this relationship by comparing repeat studies in the same animals using McNemar's test with continuity correction.

## 3 RESULTS

We performed 142 HSG examinations on 110 baboons with single examinations in 81, two in 26, and three examinations in 3 females. For the 26 females contributing two examinations, 16 had BTP observed in the first study. Among the three contributing three examinations, one had BTP only in the initial study, one only in the second examination, and one in none of the studies. BTP was confirmed in 85 (77%) of the females with the initial HSG, 12 (11%) with the second HSG, and by histologic evaluation in 10 (9%). Baseline tubal patency could not be confirmed in three females, as they received an active treatment following the initial HSG and histology demonstrated bilateral (n = 2) or unilateral (n = 1) occlusion changes consistent with the treatment effect.^8^ As all three of these females had a history of a recent term pregnancy, we considered the HSG results consistent with FO.

Table 1 provides details of the characteristics of the HSG examinations. In 33 (23%), one or both tubes were occluded (FO). Over 90% of the FO examinations occurred when serum estradiol concentrations were below 60 pg/mL. Examinations with FO were also significantly more likely to be associated with high pressure during contrast infusion (48.5 vs 3.7%) extensive vascular uptake (36.4 vs 1.8%) and difficult assessments (81.8 vs 7.3%).

**Table 1:** Findings at hysterosalpingogram examination

N (%)	Functional occlusion (n = 33)	Bilateral patency (n = 109)	*P* a
Estradiol			
≥ 60 pg/mL	3 (9.1)	46 (42.2)	.0003
< 60 pg/mL	30 (90.9)	63 (57.8)	
Subjective pressure			
Low	10 (30.3)	94 (86.2)	<.0001
Mod	7 (21.2)	11 (10.1)	
High	16 (48.5)	4 (3.7)	
Contrast (mL)			
<5	27 (81.8)	92 (84.4)	.94
5‐10	5 (15.2)	14 (12.8)	
>10	1 (3.0)	3 (2.8)	
Vascular uptake			
None	18 (54.6)	94 (86.2)	<.0001
Unilateral	3 (9.1)	13 (11.9)	
Extensive	12 (36.4)	2 (1.8)	
Difficult of assessment			
Easy	2 (6.1)	82 (75.2)	<.0001
Moderate	4 (12.1)	19 (17.4)	
Difficult	27 (81.8)	8 (7.3)	

Functional Occlusion = unilateral or bilateral tubal occlusion.

a Fisher's exact test.

**Figure 1:**
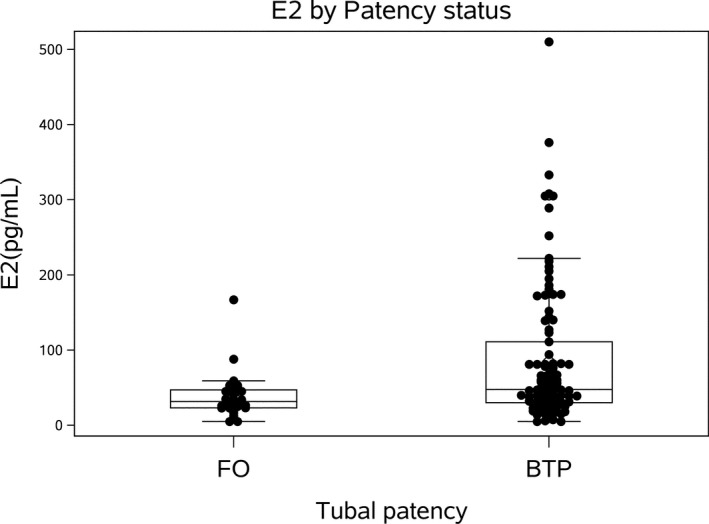
Distributionof estradiol (E_2_) concentrations in hysterosalpingogram examinations with functional occlusion (FO) and bilateral tubal patency (BTP). The median E_2_ in studies with bilateral patency (49 pg/mL) was significantly higher than in those studies with unilateral or bilateral occlusion (32 pg/mL,* P* = .005, Mann‐Whitney U‐test)

**Figure 2:**
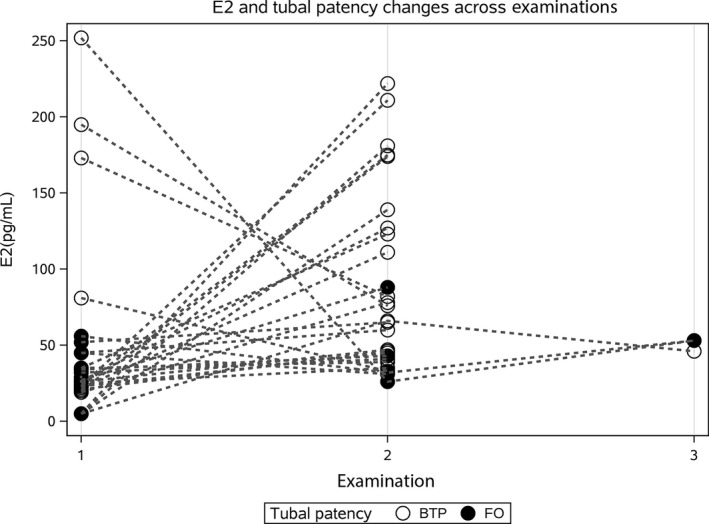
Estradiol (E_2_) levels at the time of hysterosalpingogram examination in 26 animals that underwent two, and 3 females that underwent 3 repeated studies. Closed (solid black) markers indicates examination with bilateral tubal patency (BTP), and open (white) markers an examination with functional occlusion (FO). An increase in E_2_ from < 60 pg/mL to ≥ 60 pg/mL typically resulted in a change from bilateral or unilateral occlusion to bilateral tubal patency. Similarly, a drop in E_2_ in this range resulted in a change from bilateral tubal patency to functional occlusion in some animals supporting that the change in patency was not a function of the repeat examination

**Figure 3:**
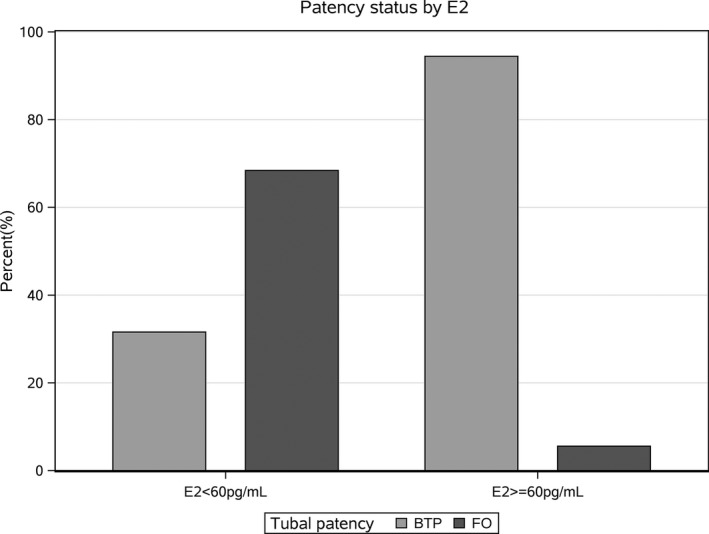
Repeat examinations in 18 females where E2 changed from <60 to ≥ 60 pg/mL. Of these, 13 results changed from functional occlusion (FO) to bilateral tubal patency (BTP). In contrast, no sets showed a change from bilateral patency to occlusion with an increase in estradiol (McNemar's Test *P* = .0001)

Figure 1 shows the distribution of estradiol concentrations as a function of tubal patency. The median E_2_ in examinations with BTP (49 pg/mL) was significantly higher than in those studies with FO (32 pg/mL, *P* = .005, Mann‐Whitney U‐test). Figure 2 shows the change in E_2_ seen with 26 second and 3 third HSG examinations. In 18 animals, 22 repeat HSG were performed with a change in E_2_ concentration from <60 to ≥ 60 pg/mL between examinations. Of these, 8 sets showed BTP with both levels of E_2_, 13 results changed from FO to BTP with the increase, none changed from BTP to FO, and one remained FO in both studies (Figure 3, McNemar's Test *P* = .0001). The animal that remained FO participated in a contraceptive study as a control and did not get pregnant over 6 cycles of exposure to a fertile male.^7^ Two other females showed FO with E_2_ > 60 pg/mL; technical difficulties occurred with both examinations.

A total of 12 females underwent examinations during the luteal phase; one periovulatory (E_2_ > 100 pg/mL, P > 1 ng/mL), 9 in the early‐to‐midluteal (E_2_ 40—100 pg/mL, P 1—10 ng/mL), and 2 in the late luteal/premenstrual (E_2_ < 40 pg/mL, P < 2 ng/mL) phase. BTP was observed in all of these studies except the two late luteal examinations (Table 2).

**Table 2:** Estradiol (E2) and progesterone (P) concentrations in females examined in the luteal phase of natural cycles

Animal	E_2_ (pg/mL)	P (ng/mL)	Patency	Cycle phase
12XX6	333	1.79	BTP	PO
18XX6	51	2.37	BTP	EML
26XX0	58	2.47	BTP	EML
34XX5	50	2.52	BTP	EML
11XX7	68	3.01	BTP	EML
27XX7	45	3.54	BTP	EML
27XX2	40	3.89	BTP	EML
13XX0	49	8.33	BTP	EML
11XX1	47	1.27	BTP	EML
19XX2	47	1.29	BTP	EML
29XX6	20	1.40	FO	LL
35XX0	35	1.50	FO	LL

Cycle phase categorized as periovulatory (PO, E_2_ > 100 pg/mL, P < 2 ng/mL), early‐to‐mid luteal (EML, E_2_ 40—100 pg/mL, P 1—10 ng/mL), or late luteal/premenstrual (LL, E_2_ < 40 pg/mL, P < 2 ng/mL). Functional Occlusion (FO) = unilateral or bilateral tubal occlusion. Bilateral tubal patency (BTP).

Hormonal therapies prior to the HSG affected estradiol levels (Table 3). GnRH agonist/antagonist therapy resulted in the greatest suppression of E_2_ (median = 23.2 pg/mL) with no significant difference between examinations showing BTP (18 pg/mL, n = 9) and FO (30 pg/mL, n = 4, *P* = .19). In contrast, animals pre‐treated with DMPA showing BTP had significantly higher E_2_ (60.5 pg/mL, n = 2) than those with FO (24.5 pg/mL, n = 8, p = .049). The highest median E_2_ (41 pg/mL) occurred in the COC‐treated group, with no significant difference between those with bilateral patency (41 pg/mL, n = 18) and tubal occlusion (41 pg/mL, n = 8, *P* = .59). All of the COC‐treated animals examined after an 8‐day hormone‐free interval showed BTP.

**Table 3:** Median estradiol (IQ range) concentrations (pg/mL) in baboons at the time of HSG examination

Pre‐treatment	All	N	BTP	N	FO	*N*	*P* a
None	45.5 (30, 67)	74	48 (30, 78)	62	44 (28, 52.5)	12	.33
GnRH agonist/antagonist	23.2 (14, 34)	13	18 (12, 25)	9	30 (24.9, 34.5)	4	.19
DMPA	26 (16, 33)	10	60.5 (40, 81)	2	24.5 (14, 27.5)	8	.049
COC	41 (21, 53)	25	41 (21, 59)	18	41 (5, 53)	7	.59
All	45 (26, 81)	142	49 (30, 123)	109	32 (23.2, 49)	33	.005

Some animals received pre‐treatment with a GnRH agonist/antagonist, DMPA, or a COC followed by a hormone‐free interval. Functional Occlusion (FO) = unilateral or bilateral tubal occlusion. Bilateral tubal patency (BTP).

a Mann‐Whitney U‐test.

## 4 DISCUSSION

In this study, we performed HSG examinations in females during various stages of normal menstrual cycles and following hormonal treatments. Although bilateral patency was seen with most (76.7%) studies, we found a significantly lower median concentration of estradiol in the group with functional occlusion with 90% of these results associated with an estradiol concentration < 60 pg/mL. We also found low estradiol associated with a subjective impression of high uterine pressure, difficult examinations, and extravasation of contrast media into the vascular system. Furthermore, when females shown to have FO in an examination with an estradiol < 60 pg/mL underwent a repeat examination with an estradiol > 60 pg/mL, BTP was typically observed. In a limited group of studies performed in the luteal phase, we observed no independent effect of progesterone; FO occurred only in association with low levels of estradiol in the late luteal phase.

Strengths of our study include the large number of animals studied, and the subset that underwent repeat examinations allowing us to evaluate the effect of different hormonal conditions. Additionally, we verified bilateral tubal patency either by HSG or by histology in all but three of the subjects. However, these 3 each had a history of a recent confirmed pregnancy, strongly suggestive of tubal patency. Major limitations include the retrospective nature of our data analysis, the lack of strict timing of hormonal treatments, and the absence of a true experimental approach to evaluate estrogen action. As we did not perform examinations at well‐controlled time points in the menstrual cycle, we cannot provide information as to the sensitivity and specificity of the HSG examination in baboons in the follicular or luteal phase.

The baboon provides an excellent model for reproductive research as the reproductive tract is similar to women and influenced by the same hormonal events.^11^ Unlike most macaque species, the baboon has a cervix with a straight path similar to women that permits routine dilation and intrauterine procedures including hysterosalpingography. However, a notable species difference in baboons is dramatic perineal sex skin swelling that peaks with ovulation prior to regressing.^10^ In an effort to standardize and facilitate our evaluation of animals, we treated some females with DMPA or a GnRH agonist/antagonist prior to the HSG examination. Difficultly confirming tubal patency in these females led us to explore the hypothesis that low concentrations of estradiol may contribute to functional occlusion.

Although GnRH agonists/antagonists resulted in the lowest concentrations of estradiol, a higher proportion of females pre‐treated with DMPA developed FO. This suggests that a progesterone receptor‐mediated effect contributes to regulation of the UTJ, perhaps through downregulation of estrogen receptor.^12^, ^13^ We observed FO in all of the females pre‐treated with DMPA >7 day prior to HSG, but BTP with shorter exposure. These results are similar to those observed with progesterone exposure during natural cycles, with FO seen only in the late luteal phase.

Although we observed a correlation between an estradiol concentration of < 60 pg/mL and FO, most HSG examinations performed even with low levels of estradiol showed BTP and three females with E_2_ > 60 had FO suggesting that multiple factors influence patency at the UTJ. Prior research investigating the nature of proximal tubal obstruction observed by HSG in women has suggested two mechanisms. Based on a review of cases of normal histology of the intramural tube following cornual resection, Sulak et al^14^ proposed that amorphous plugs (of undescribed origin) form a cast that obstructs the tube. Without presenting new data, Papaioannou expanded on this idea hypothesizing that high smooth muscle tone and reduced ciliary activity during the follicular phase result in increased tubal secretions at the utero‐tubal junction (UTJ) leading to stasis of the luminal contents and functional obstruction.^3^ Sulak and Papaioannou's hypothesis has not been confirmed and seems unlikely given the need for sperm to gain access to the upper reproductive tract during the late follicular phase for normal fertility, and non‐human primate data demonstrating that ciliary activity increases in response to estrogen.^15^

Muscular contractility offers a better explanation. The UTJ consists of three different smooth muscle layers: inner longitudinal, circular, and outer uterine spiral. Korenaga and Kadota found a high contractile response in isthmic fallopian tube circular muscle obtained during the proliferative phase, but an inhibitory response in secretory phase samples.^16^ This may explain why some animals show a hormonal sensitivity leading to functional occlusion. Wilhelmsson and colleagues mechanically separated these layers, and determined that administration of prostaglandin (PG) E2 stimulated contraction of the uterine layer.^17^

We recently performed repeated HSG examinations in 10 women during the follicular and luteal phase of normal menstrual cycles, and 30 days after treatment with a combined pill and DMPA. All 10 subjects demonstrated BTP on at least one HSG examination during the study. One subject showed bilateral FO during the follicular phase examination (E_2_ = 36 pg/mL), but BTP in the luteal phase (E_2_ = 52 pg/mL), COC cycle, and DMPA examinations. Two subjects had functional occlusion during the COC cycle, and one following treatment with DMPA.^18^ These results suggest that our findings in the baboon apply to human medicine.

Hormonal regulation of muscular tone at the UTJ may offer selective advantages. The female genital tract provides a direct pathway from the outside world to the peritoneal cavity. Evolution would favor a strategy designed to prevent the entry of pathogens into the upper genital tract, while accommodating the essential functions of reproduction. A hormonally mediated blockade of the UTJ could represent an evolutionary adaptation to reduce the risk of endometriosis or ascending infection. We hypothesize that the UTJ relaxes in response to rising concentrations of estradiol in the mid‐to‐late follicular phase to allow sperm to enter the fallopian tubes prior to ovulation and remains fully open through the mid‐luteal phase to allow time for the fertilized embryo to enter the uterine cavity. In the late luteal phase, in response to progesterone receptor‐mediated downregulation of estrogen receptor and declining estradiol levels, the adaptive strategy would favor closure of the UTJ to prevent retrograde menstruation. This balance between reproductive and protective functions of the UTJ should result in the expression of a variety of phenotypes. An imbalance leading to prolonged closure of the UTJ could result in infertility while the tendency toward prolonged patency could increase the risk of endometriosis.

In conclusion, our results demonstrate an association between functional occlusion of the fallopian tube and low serum estradiol concentration in baboons, with 90% of cases of FO seen with E_2_ < 60 pg/mL. Furthermore, among females with findings of FO during an examination with E_2_ < 60 pg/mL, a repeat HSG examination conducted with E_2_ ≥ 60 pg/mL resulted in BTP. Together, these findings suggest that hysterosalpingography or transcervical permanent contraception procedures should be performed during the mid‐to‐late follicular phase to ensure adequate estradiol exposure to avoid a false diagnosis of functional fallopian tube occlusion or incomplete procedures.

## CONFLICTS OF INTEREST

None of the authors have financial conflicts of interest related to this research.
